# Increased interleukin-1β levels following low dose MDMA induces tolerance against the 5-HT neurotoxicity produced by challenge MDMA

**DOI:** 10.1186/1742-2094-8-165

**Published:** 2011-11-24

**Authors:** Andrea Mayado, Elisa Torres, Maria D Gutierrez-Lopez, Maria I Colado, Esther O'Shea

**Affiliations:** 1 Departamento de Farmacologia, Facultad de Medicina, Universidad Complutense, Madrid, Spain

**Keywords:** MDMA, preconditioning, tolerance, IL-1β, IL-1ra, sIL-1RI, neurotoxicity, 5-HT

## Abstract

**Background:**

Preconditioning is a phenomenon by which tolerance develops to injury by previous exposure to a stressor of mild severity. Previous studies have shown that single or repeated low dose MDMA can attenuate 5-HT transporter loss produced by a subsequent neurotoxic dose of the drug. We have explored the mechanism of delayed preconditioning by low dose MDMA.

**Methods:**

Male Dark Agouti rats were given low dose MDMA (3 mg/kg, i.p.) 96 h before receiving neurotoxic MDMA (12.5 mg/kg, i.p.). IL-1β and IL1ra levels and 5-HT transporter density in frontal cortex were quantified at 1 h, 3 h or 7 days. IL-1β, IL-1ra and IL-1RI were determined between 3 h and 96 h after low dose MDMA. sIL-1RI combined with low dose MDMA or IL-1β were given 96 h before neurotoxic MDMA and toxicity assessed 7 days later.

**Results:**

Pretreatment with low dose MDMA attenuated both the 5-HT transporter loss and elevated IL-1β levels induced by neurotoxic MDMA while producing an increase in IL-1ra levels. Low dose MDMA produced an increase in IL-1β at 3 h and in IL-1ra at 96 h. sIL-1RI expression was also increased after low dose MDMA. Coadministration of sIL-1RI (3 μg, i.c.v.) prevented the protection against neurotoxic MDMA provided by low dose MDMA. Furthermore, IL-1β (2.5 pg, intracortical) given 96 h before neurotoxic MDMA protected against the 5-HT neurotoxicity produced by the drug, thus mimicking preconditioning.

**Conclusions:**

These results suggest that IL-1β plays an important role in the development of delayed preconditioning by low dose MDMA.

## Background

3,4- Methylenedioxymethamphetamine (MDMA or "ecstasy"), an amphetamine derivative, is a popular drug of abuse among young people. In experimental animals, MDMA produces a series of immediate neurochemical, biochemical and behavioural effects as well as producing long-term species-specific neurotoxicity [1]. In rats, MDMA produces an apparent loss of 5-HT nerve terminals [2,3] demonstrated by immunohistochemical techniques and biochemically the damage is reflected by a substantial decrease in the concentration of 5-HT and its metabolite, 5-hydroxyindolacetic acid (5-HIAA) [4,5] and a reduction in the density of 5-HT uptake sites labelled with [^3^H]-paroxetine [4,6,7]. MDMA produces a hyperthermic response immediately after injection which lasts at least 5-6 h and appears to modulate the long-term neuronal damaged caused by the drug [8,9]. MDMA also produces a neuroinflammatory response characterised by an increase in mature IL-1β and of its precursor protein (pro-IL-1β) in rat frontal cortex as well as microglial activation [9,10].

When exposed to practically any stimulus capable of causing injury at a level close to (but below) the threshold of damage, most living organisms respond with protective mechanisms to potentially recurring challenges. Janoff introduced the term "preconditioning" for this phenomenon [11]. The preconditioning phenomenon has been observed in different tissues and organs including the brain, and in different models of injury. Pre-exposure to heat shock treatment, mild ischemia or hypoxia induces tolerance against a subsequent neurotoxic insult in rodents [12-16]. The development of preconditioning may involve a number of different effectors including the activation of cellular defence mechanisms such as antioxidant systems, heat shock proteins and cell death/survival determinants, or responses at tissue level for example reduced inflammatory responsiveness [17]. In line with this ischemic tolerance has been shown to involve IL-1β and IL-1ra [12,18,19]. Repeated exposure to heat shock offers protection against MDMA-induced 5-HT neurotoxicity in Dark Agouti rats [13] and more recently it has been established that prior exposure of adult rats to MDMA provides protection against a subsequent MDMA-induced 5-HT depletion in the brain [20,21]. The neuroprotection exerted is independent of the ambient temperature at which the low dose MDMA is given [21] and does not appear to involve the hyperthermic response, alterations in brain MDMA pharmacokinetics or changes in 5-HT transporter activity [20, 21, unpublished observations].

IL-1β is often described as the prototypical pro-inflammatory cytokine. Released in response to local or systemic injury or disease, IL-1β orchestrates host defence response. It is a potent pyrogen [22] and a key mediator of innate and adaptive immune response [23]. IL-1β exerts its action by binding of IL-1 type I receptor (IL-1RI), forming a complex which then binds to the IL-1R accessory protein (IL-1RAcP), resulting in the initiation of signal transduction [24]. IL-1 receptor antagonist (IL-1ra) is a competitive antagonist of IL-1RI that selectively binds, but fails to trigger receptor association with the accessory protein resulting in the blockade of all known actions of IL-1. The extracellular domain of IL-1RI may also be proteolytically cleaved from the cell surface giving rise to IL-1 soluble type I receptor (IL-1sRI) [25]. This soluble form of the receptor binds both IL-1β and IL-1ra but does not initiate signal transduction.

The aims of this study were: 1) to describe MDMA preconditioning (interval, number of doses), 2) to examine the time-course of low dose MDMA-induced changes in IL-1β, IL-1ra levels and soluble and membrane bound IL-1RI expression in rat frontal cortex, 3) to study the effect of exogenously administered sIL-1RI on the protection induced by MDMA pre-treatment against subsequent neurotoxic MDMA, and 4) to analyse the effect of exogenously administered IL-1β against subsequent neurotoxic MDMA.

We have demonstrated that low dose MDMA can provide long-lasting neuroprotection against a subsequent challenge of higher dose MDMA and that the preconditioning appears to involve low dose MDMA-induced IL-1β release.

## Methods

### Animals, drugs and reagents

Male Dark Agouti rats (175-200 g, Harlan Laboratories Models, Barcelona) were used. MDMA induces a reproducible acute hyperthermic response in this strain [5,26] and also a reproducible long-term neurotoxic loss of 5-HT terminals after a single dose [5]. Rats were housed in groups of 5 in conditions of constant temperature (21°C ± 2°C) and a 12 h light/dark cycle (lights on: 08 h 00 min) and given free access to food and water. Low dose MDMA (3 mg/kg, i.p.) was given once or repeatedly (1 injection daily for 4 days) to rats and 24 h, 96 h or 7 days later animals received neurotoxic MDMA (12.5 mg/kg, i.p.). Room temperature at the time of MDMA administration was 21-22°C. To determine neurotoxicity, [^3^H]-paroxetine-labelled 5-HT transporter density was measured in frontal cortex 7 days after neurotoxic MDMA. To determine IL-1β and IL-1ra levels and IL-1RI expression, animals were killed 3 h, 6 h, 24 h or 96 h after MDMA (3 mg/kg, i.p.) or 1 h or 3 h after receiving neurotoxic MDMA.

MDMA hydrochloride (LIPOMED, Arlesheim, Suiza) was dissolved in saline (0.9% NaCl) and given in a volume of 1 mL/kg. The dose is reported in terms of the base. Control animals were injected with saline.

For intracerebroventricular (i.c.v.) and intracortical administrations rats were anesthetized with a mixture of isoflurane (3.5% for induction, 1-2% for maintenance; flow rate 1.5 L min^-1^) and nitrous oxide/oxygen mixture (30/70%) in air and placed in a stereotaxic frame secured in a Kopf stereotaxic frame with the tooth bar 3.3 mm below interaural zero. A 22 G guide cannula was implanted in the right lateral ventricle or frontal cortex (according to the following coordinates: for ventricle: 7.9 mm rostral to the interaural line, 0.8 mm lateral to the midline and 3.1 mm below the skull surface; and for cortex: 8.9 mm rostral to the interaural line, 0.4 mm lateral to the midline and 2.1 mm below the skull surface [27]. The cannula was secured to the skull as described by Baldwin *et al*. [28]. Injections took place 5 days after surgery through a 28 G injector (Plastics One, Roanoke, VA, USA) which fitted in and protruded 1 mm beyond the guide cannula.

Soluble interleukin-1 receptor type I (sIL-1RI; Sigma-Aldrich, Madrid, Spain) and recombinant rat IL-1β (R&D Systems, Minneapolis, MN, USA) were dissolved in phosphate-buffered saline (PBS) containing 0.1% bovine serum albumin (BSA). sIL-1RI (3 μg in 5 μL of PBS over 5 min; i.c.v.) was administered 5 min before and 3 h after low dose MDMA. IL-1β (2.5 pg in 5 μL over 5 min; intracortical) was administered 96 h before neurotoxic MDMA.

All experimental procedures were performed in accordance with the guidelines of the Animal Welfare Committee of the Universidad Complutense de Madrid (following European Council Directives 86/609/CEE and 2003/65/CE).

### Measurement of rectal temperature

Immediately before and up to 6 h after MDMA injection, temperature was measured by use of a digital readout thermocouple (BAT12 thermometer, Physitemp, NJ, USA) with a resolution of 0.1°C and accuracy of ± 0.1°C attached to a RET-2 Rodent Sensor which was inserted 2.5 cm into the rectum of the rat, the animal being lightly restrained by holding it in the hand. A steady readout was obtained within 10 s of probe insertion.

### Western blot for IL-1RI immunoreactivity

Expression of IL-1RI was determined in the soluble and membrane fractions of frontal cortex by Western blot. Preparation of soluble and membrane fractions was carried out by modification of the method described by Wang *et al*. [29]. Tissue was homogenized by sonication in ice-cold buffer contain 50 mM Tris, 320 mM sucrose and a number of phosphatase and protease inhibitors (0.2 M phenylmethanesulphonylfluoride, 0.5 M NaVO_4_, 1 M NaMoO_4 _and Complete Mini, Roche, Spain). Samples were centrifuged at 100 000 × g for 60 min at 4°C and protein determined in the supernatant [30]. This was the fraction containing the soluble form of IL-1RI. The pellet was resuspended in the same buffer by sonication. The homogenate was centrifuged 12 000 × g for 20 min at 4°C and protein determined in the supernatant [30]. This was the fraction containing the membrane bound form of IL-1RI.

The samples were boiled in Laemmli buffer and aliquots containing 100 μg of protein were separated by 10% SDS-polyacrylamide gel electrophoresis (SDS-PAGE) and transferred to nitrocellulose membranes. Nonspecific sites were blocked by incubation for 1 h in TBS containing 5% skimmed milk. Membranes were incubated overnight at 4°C with monoclonal rabbit anti IL-1RI (Abcam, UK; 1:1000) as primary antibody followed by incubation with anti-rabbit IgG-horseradish peroxidase (GE Healthcare, Spain; 1:2000) for 2 h. Equal protein sample loading was confirmed by quantification of the β-actin signal.

Immunoreactivity was detected with an enhanced chemiluminescence Western blot detection system (GE Healthcare) followed by exposure to Amersham Hyperfilm ECL (GE Healthcare) for 1-10 min. Different film exposure times were used to ensure that band were not saturated. Quantification of specific bands on the film was performed using the Quantity One program (BioRad Laboratories, Inc, CA, USA). Each IL-1RI band density was normalised for protein content by referring it to its β-actin band density and then the IL-1RI expression in the different experimental conditions was expressed as a percentage of the control group.

### IL-1β and IL-1ra immunoassays

Brain levels of IL-1β and IL-1ra were determined in frontal cortex using commercially available sandwich enzyme-linked immunosorbent assays (ELISA) (Rat IL-1β/IL-1F2 and Human IL-1ra/IL-1F3 Quantikine ELISA Kits, respectively; R&D Systems, Minneapolis, MN, USA). According to the manufacturer, the IL-1β kit provides a valid measure of the levels of mature 17 kDa IL-1β (the limit of sensitivity was 5 pg/mL) but underestimates the precursor form 31 kDa IL-1β (non-biologically active). Samples were prepared by homogenization in 5 volumes of ice-cold buffer (50 mM Tris, 320 mM sucrose, 1 mM dithiothreitol, 10 μg/mL leupeptin, 2 μg/mL aprotinin and 0.2% phenantroline; pH 7.0). Samples were centrifuged at 14 000 × g for 20 min at 4°C. Protein was determined in the supernatant [30]. Samples were assayed in triplicate following the manufacturer's guidelines. Quantification was performed using a standard curve of increasing concentrations of IL-1β or IL-1ra. The optical density of each well was determined using a microplate reader (ELX808 IU, Ultra Microplate Reader, Bio-Tek Instruments, VT, USA) set to 450 nm (correction wavelength set at 540 nm). Intra-assay and inter-assay variations were less than 5% and 15%, respectively, for both kits.

### Quantification of 5-HT transporter density by [^3^H]-paroxetine binding

[^3^H]-Paroxetine binding was measured by the method described in detail by Hewitt and Green [6]. Briefly, frontal cortex from individual animals was homogenized in ice-cold Tris-HCl (50 mM; pH 7.4) containing NaCl (120 mM) and KCl (5 mM) using an Ultra-Turrax. The homogenate was centrifuged at 30 000 × g for 10 min at 4°C. The supernatant was discarded and the wash procedure repeated twice more. The pellet was finally resuspended in the Tris buffer at a concentration of 10 mg tissue/mL. Aliquots of tissue (800 μL) were incubated with a saturating concentration of [^3^H]-paroxetine (1 nM, specific activity = 21.4 Ci/mmol, Perkin-Elmer, Spain) for 90 min at room temperature in the absence and presence of 5-HT (100 μM) for determination of total and non-specific binding, respectively. Assays were terminated by rapid filtration through glass fibre filters and radioactivity determined by scintillation spectrometry. Protein was determined by the method of Lowry *et al*. [31].

### Statistics

Data from ELISA, immunoreactivity and 5-HT transporter studies were analyzed using one-way ANOVA followed by Newman-Keuls multiple-comparisons test when a significant *F *value was obtained. Statistical analyses of temperature measurements were performed by two-way ANOVA with repeated measures using treatment as the between subjects factor and time as the repeated measure, followed by Bonferroni as post-test (GraphPad Prism 5; GraphPad Software Inc., San Diego, CA, USA).

## Results

### Effect of pretreatment with low dose MDMA against MDMA-induced loss of 5-HT uptake sites in frontal cortex

To study the effect of pretreatment with low dose MDMA on the loss of 5-HT uptake sites (5-HTT) produced by a neurotoxic dose of MDMA, rats were treated with a daily dose of MDMA (3 mg/kg, i.p.) for 4 consecutive days and given MDMA (12.5 mg/kg, i.p.) 24 h after the final dose. Separate groups of animals were given a single MDMA (3 mg/kg, i.p.) injection 24 h, 96 h or 7 days before MDMA (12.5 mg/kg, i.p.) administration.

**Figure 1:**
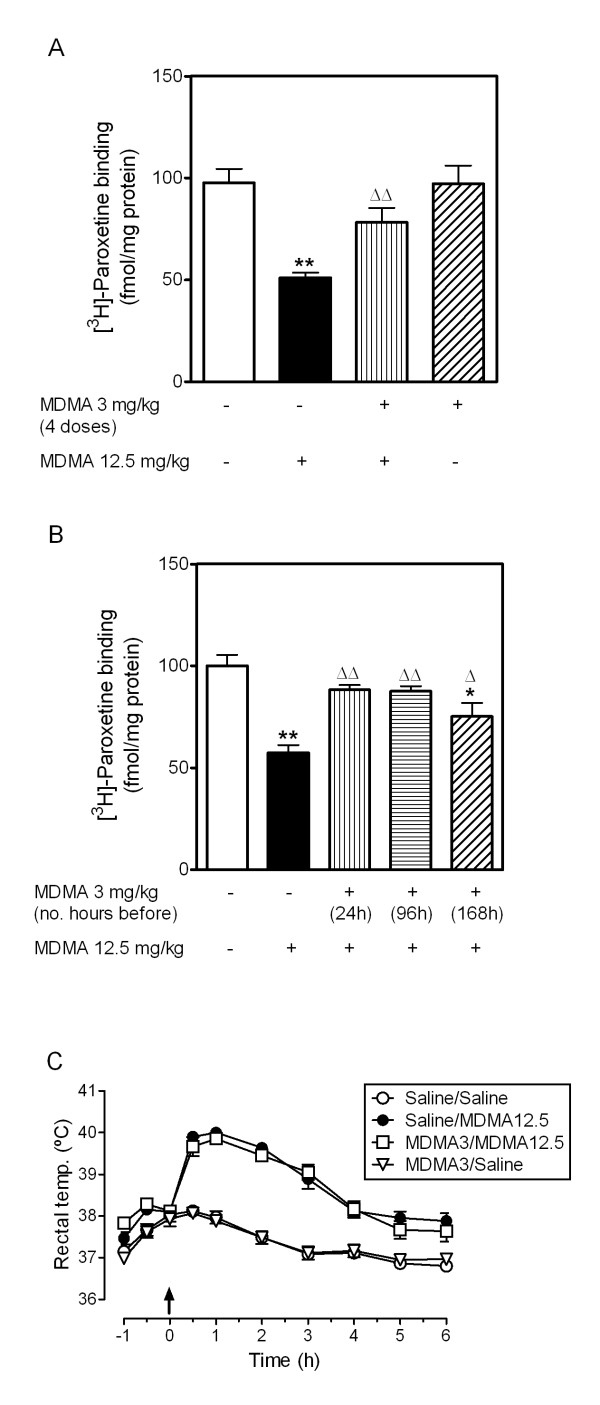
**Effect of pretreament with low dose MDMA (3 mg/kg, i.p.) on MDMA (12.5 mg/kg, i.p.)-induced neurotoxicity in frontal cortex 7 days later**. (A) Pretreatment with four consecutive doses of low dose MDMA attenuated the loss of 5-HT transporters induced by neurotoxic MDMA given 24 h later. (B) Pretreatment with a single low dose of MDMA attenuated the loss of 5-HT transporters induced by neurotoxic MDMA given 24 h, 96 h or 168 h (7 days) later. (C) Pretreatment with low dose MDMA 96 h earlier did not modify neurotoxic MDMA-induced hyperthermia. The arrow marks the time of neurotoxic MDMA injection. Results shown as mean ± s.e.mean, n = 5-8. Different from saline-treated controls: *P < 0.05, **P < 0.001. Different from neurotoxic MDMA: ^Δ^P < 0.05, ^ΔΔ^P < 0.01. One-way ANOVA followed by Newman-Keuls test.

Pretreatment with repeated low dose MDMA attenuated the decrease in 5-HTT density induced by a subsequent neurotoxic dose of MDMA in the frontal cortex (58% protection) but did not modify the density of 5-HT uptakes sites in saline treated-animals (Figure 1A). Administration of a single low dose MDMA injection 24 h or 96 h before neurotoxic MDMA also prevented the loss of 5-HTT (Figure 1B). The level of protection was similar to that observed following repeated doses and similar for both intervals (71-72%), however, when low dose MDMA was given 7 days before neurotoxic MDMA, the protection provided was less marked (42%; Figure 1B).

### Effect of pretreatment with low dose MDMA on neurotoxic MDMA-induced hyperthermia

Two-way ANOVA indicated that there was a significant effect of treatment (F_3,20 _= 104.4, P < 0.0001), time (F_9,180 _= 87.0, P < 0.0001) and interaction (F_27,180 _= 9.48, P < 0.0001). Bonferroni post test revealed that MDMA (12.5 mg/kg, i.p.) produced a hyperthermic response which peaked 60 min after treatment and was sustained for up to 6 h after treatment. Pretreatment with a single low dose of MDMA (3 mg/kg, i.p.) 96 h earlier did not affect the hyperthermia of neurotoxic MDMA or the rectal temperature of saline-treated animals (Figure 1C).

Since a single low dose of MDMA administered 96 h before neurotoxic MDMA provided extensive delayed neuroprotection this protocol was used for further studies.

### Effect of pretreatment with low dose MDMA on neurotoxic MDMA-induced changes in IL-1β and IL-1ra levels in frontal cortex

**Figure 2:**
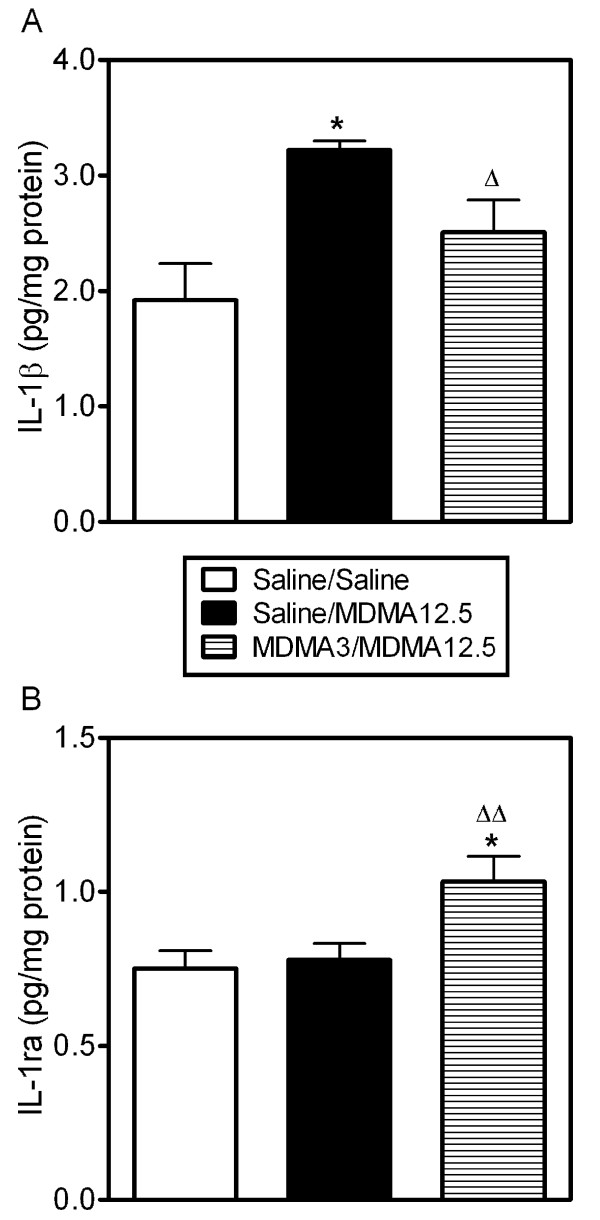
**Effect of pretreatment with a single low dose of MDMA (3 mg/kg, i.p.) 96 h before on MDMA (12.5 mg/kg, i.p.)-induced changes in IL-1β and IL-1ra levels in frontal cortex**. (A) Pretreatment with low dose MDMA attenuated the increase in IL-1β produced by neurotoxic MDMA 3 h later. (B) Pretreatment with low dose MDMA produced an increase in IL-1ra levels 1 h after neurotoxic MDMA. Results shown as mean ± s.e.mean, n = 7-8. Different from saline-treated controls: *P < 0.05. Different from neurotoxic MDMA: ^Δ^P < 0.05, ^ΔΔ^P < 0.01. One-way ANOVA followed by Newman-Keuls test. Levels of the cytokines in animals treated only with low dose MDMA were not included but were considered to be similar to those observed at the 96 h time point of Figure 3.

Administration of neurotoxic MDMA (12.5 mg/kg, i.p.) produced an increase in IL-1β levels in frontal cortex 3 h after injection as previously described [9,32]. Pretreatment with a single low dose of MDMA (3 mg/kg, i.p.) 96 h before giving the neurotoxic dose attenuated this increase in IL-1β (Figure 2A).

Neurotoxic MDMA did not modify IL-1ra levels in frontal cortex as described previously [33], however pretreatment with low dose MDMA produced an increase in IL-1ra levels 1 h after the neurotoxic dose (Figure 2B).

### Effect of low dose MDMA on IL-1β and IL-1ra levels in frontal cortex

**Figure 3:**
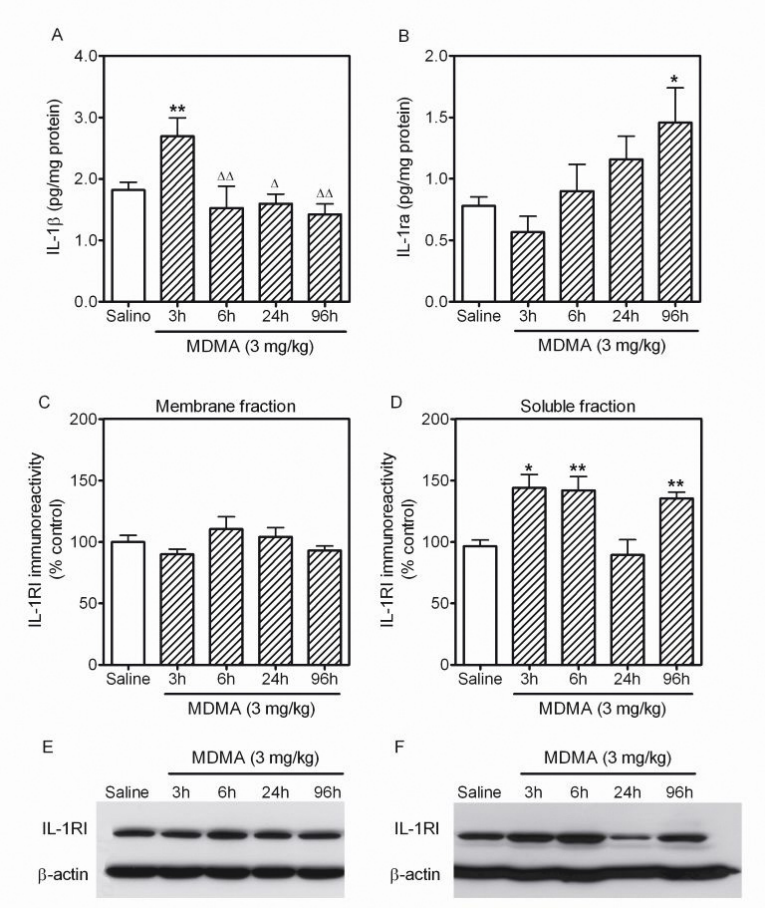
**Effect of low dose MDMA (3 mg/kg, i.p.) on (A) IL-1β and (B) IL-1ra levels and IL-1RI expression in the membrane (C) and soluble (D) fractions of frontal cortex**. MDMA produced 1 h later an increase in IL-1β levels (A) and 96 h later an increase in IL-1ra (B). IL-1RI expression was unaltered in the membrane fraction (C) but increased in the soluble fraction 3 h, 6 h and 96 h after MDMA (D). (E) and (F) are representative images from the Western blots of membrane (E) and soluble (F) fractions of frontal cortex. Results shown as mean ± s.e.mean, n = 4-8. Different from saline-treated controls: *P < 0.05, **P < 0.01. Different from neurotoxic MDMA: ^Δ^P < 0.01, ^ΔΔ^P < 0.001. One-way ANOVA followed by Newman-Keuls test.

For the examination of IL-1β and IL-1ra levels following low dose MDMA, animals were killed 3 h, 6 h, 24 h and 96 h after an injection of MDMA 3 mg/kg. Administration of a single low dose of MDMA produced an increase in IL-1β levels in the frontal cortex 3 h after injection compared with the saline-treated control group (Figure 3A). However, no changes in levels were observed 6 h, 24 h or 96 h after injection.

A single low dose of MDMA produced an increase in IL-1ra levels in the frontal cortex 96 h after administration without producing significant changes in the levels at earlier time points (Figure 3B).

### Effect of low dose MDMA on IL-1 receptor type I (IL-1RI) expression in frontal cortex

IL-1RI expression in the membrane and soluble fractions of frontal cortex was measured 3 h, 6 h, 24 h and 96 h after low dose MDMA administration.

MDMA did not alter IL-1RI expression in the membrane fraction at any of the time-points examined (Figure 3C). However, in the soluble fraction a biphasic response was observed. IL-1RI expression increased 3 h and 6 h after MDMA, returned to control levels at 24 h and rose again at 96 h (Figure 3D).

### Effect of sIL-1RI on the protection provided by low dose MDMA against neurotoxic MDMA

**Figure 4:**
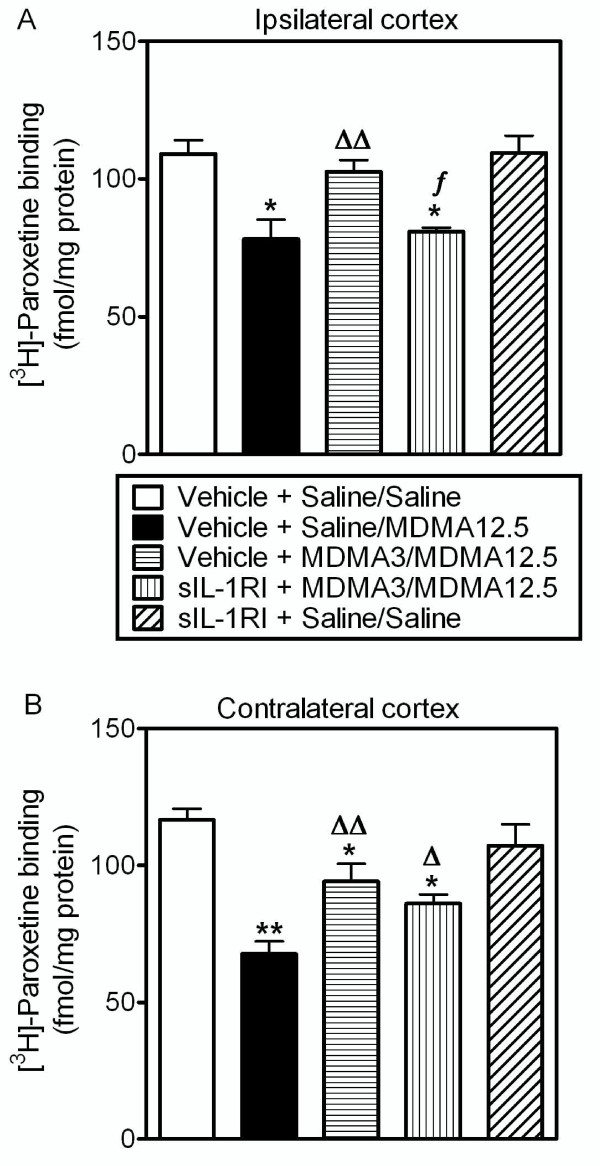
**Effect of sIL-1RI given with low dose MDMA (3 mg/kg, i.p.) on the protection against MDMA (12.5 mg/kg, i.p.)-induced loss of 5-HT transporters in ipsilateral (A) and contralateral (B) frontal cortex 7 days later**. Neurotoxic MDMA reduced the number of 5-HT transporters in both sides of the frontal cortex and this was prevented by low dose MDMA given 96 h earlier. Administration of sIL-1RI (3 μg, i.c.v.) 5 min before and 3 h after low dose MDMA prevented this protection in ipsilateral but not in contralateral frontal cortex. Results shown as mean ± s.e.mean, n = 5-7. Different from saline-treated controls: *P < 0.01, **P < 0.001. Different from neurotoxic MDMA: ^Δ^P < 0.05, ^ΔΔ^P < 0.01. Different from MDMA3/MDMA12.5: *^f^*P < 0.05. One-way ANOVA followed by Newman-Keuls test.

MDMA (12.5 mg/kg, i.p.) produced a decrease in the density of 5-HTT in both ipsi- and contralateral frontal cortex 7 days after treatment. This 5-HTT loss was attenuated by pretreatment with MDMA (3 mg/kg, i.p.) given 96 h before the neurotoxic dose (Figure 4A, B). Injection of sIL-1RI (3 μg per animal, i.c.v.) 5 min before and 3 h after low dose MDMA prevented the neuroprotection exerted by the pretreatment in the ipsilateral cortex (Figure 4A) but failed to modify it in the contralateral side (Figure 4B). sIL-1RI administration alone did not alter MDMA neurotoxicity in the frontal cortex [33] or modify the density of 5-HTT in saline-treated controls (Figure 4A, B). Furthermore, the administration of sIL-1RI did not alter neurotoxic MDMA-induced hyperthermia (data not shown).

### Effect of pretreatment with IL-1β on the 5-HTT loss induced by neurotoxic MDMA

In order to avoid any dilution of the effects by the use of tissue which had not been reached the IL- β injection, only tissue around the injection site was used in this assay. Tissue was obtained 2 mm rostral to and caudal from the injection site using a rat brain matrix.

**Figure 5:**
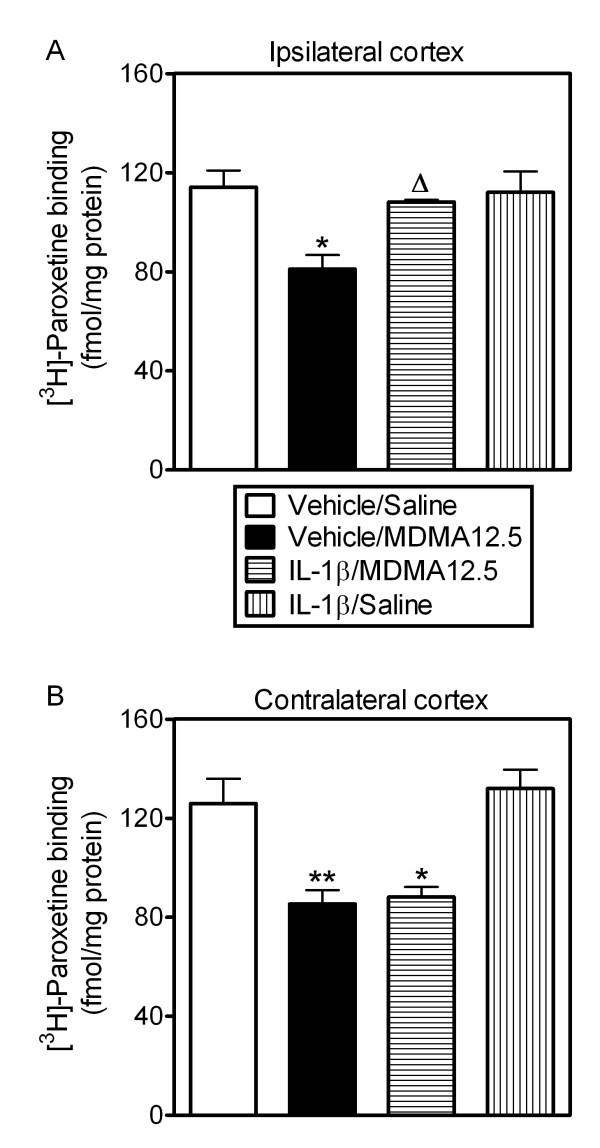
**Effect of pretreatment with IL-1β on MDMA (12.5 mg/kg, i.p.)-induced neurotoxicity in frontal cortex 7 days later**. IL-1β (2.5 pg, intracortical) given 96 h before neurotoxic MDMA prevented the loss of 5-HT transporters in the ipsilateral (A) but not in the contralateral (B) frontal cortex. Results shown as mean ± s.e.mean, n = 4-7. Different from saline-treated controls: *P < 0.05, **P < 0.01. Different from neurotoxic MDMA: ^Δ^P < 0.05. One-way ANOVA followed by Newman-Keuls test.

As described above, MDMA (12.5 mg/kg, i.p.) produced a significant reduction in the density of 5-HTT in the ipsi- and contralateral sides of the frontal cortex 7 days later (Figure 5A, B). Injection of IL-1β (2.5 pg, intracortical) 96 h before administration of neurotoxic MDMA (12.5 mg/kg, i.p.) attenuated the reduction in the density of [^3^H]-paroxetine-labelled 5-HT uptake sites (Figure 5A). In the contralateral cortex, IL-1β pretreatment did not attenuate the decrease in the density of 5-HT uptake sites induced by neurotoxic MDMA (Figure 5B).

## Discussion

Preconditioning, a phenomenon by which exposure to a non-harmful stimulus confers tolerance to subsequent exposure to a more intense stimulus of the same or of a different nature, has been described in many different tissues and organs following exposure to a diverse range of stimuli [17,34]. We have previously described a model of "cross-tolerance" in which heat-shock pretreatment protects against the 5-HT neurotoxicity induced by MDMA [13] and more recently low dose MDMA-induced tolerance has been described [20,21]. The present results indicate an important role for interleukin-1beta (IL-1β) in the development of this low dose MDMA-induced tolerance against the 5-HT neurotoxicity induced by a higher subsequent dose of the drug.

In the present study we observed that the protection produced by low dose MDMA is not dependent on the number of doses applied since a single dose of MDMA 3 mg/kg protects to a similar extent as 4 consecutive administrations against the toxicity of neurotoxic MDMA given 24 h later. The protection provided by a single low dose of MDMA is long-lasting being observed when the pretreatment is given 24 h and 96 h earlier and continuing to provide partial protection when given up to 7 days before the higher dose of MDMA. The results agree with those of Piper *et al *[35,36] who described protection against binge MDMA-induced 5-HT neurotoxicity 7 days following chronic exposure to low doses of the drug in both adolescent and adult rats but differ from those described by Bhide *et al *[20] where tolerance against a binge dosing regimen of 10 mg/kg every 2 h for 4 doses was relatively transient in nature and lost when the window was extended to 4 days. The time-scale of neuroprotection observed indicates that the processes involved in its development might involve the synthesis of new proteins which could provide a wide window for protection. Studies in other lesion models in which tolerance can be induced by exposure to a less intense stimulus have revealed two windows in which tolerance develops. The first is a rapid tolerance which is observed minutes after the trigger and lasts for hours involving factors such as K_ATP _channels, nitric oxide, adenosine and protein kinase alpha/delta [34]. A second window of protection, delayed tolerance, develops over hours and days, and usually involves *de novo *protein synthesis. Most inducers of tolerance can produce both rapid and delayed tolerance. The protection observed in this study can be described as delayed tolerance since it develops hours after the initial insult [34]. Whether or not low dose MDMA can also produce rapid tolerance is unknown. A low dose of MDMA followed by a neurotoxic dose within minutes or hours would, in all likelihood, produce a potentiated hyperthermic reaction much as is observed following the administration of low dose MDMA in binge protocols where the rectal temperature increases following each subsequent dose separated by 3 h [37]. This would result in increased neurotoxicity [38]. Separation of the low and high dose MDMA by 96 h resulted in no change in the hyperthermic response of neurotoxic MDMA and a similar lack of effect was observed with a 24 h interval [21]. This allows us to rule out changes in the hyperthermic reaction as a factor involved in the neuroprotection observed as previously described [20,21].

At doses that produce neurotoxicity, MDMA produces a neuroinflammatory reaction characterised by an increase in IL-1β levels and microglial activation in the frontal cortex [9,10,32,33,39]. A single low dose of MDMA given 96 h earlier reduces the elevation in IL-1β levels induced by neurotoxic MDMA. Although the participation of IL-1β in MDMA-induced neurotoxicity has not been thoroughly established, it appears it may play at least a partial role in its development since compounds which reduce IL-1β release and microglial activation following MDMA partially protect against 5-HT neurotoxicity [32,39].

The suppression of the increase in IL-1β levels following neurotoxic MDMA is accompanied by an increase in IL-1ra levels in the frontal cortex, an effect not observed following neurotoxic MDMA given alone [33; present results]. This increase in IL-1ra levels is due to the pretreatment with low dose MDMA rather than to an interaction between the two treatments since the increase is also observed 96 h after the pretreatment. IL-1ra, the endogenous antagonist of IL-1β, which may be released as a consequence of large increases in IL-1β levels, binds to the IL-1RI with high affinity and inhibits IL-1β signal transduction thus providing a mechanism for limiting IL-1β mediated responses [40]. In fact, IL-1ra has been shown to protect in several models of brain injury including ischemic, excitotoxic and traumatic brain insults [41-44]. The role of this antagonist in protection is further supported by studies in which inhibition or deletion of endogenous IL-1ra enhances ischemic brain injury [45] and increases inflammatory responses [46]. In addition, endogenous IL-1ra has been implicated in the neuroprotective effects of cannabinoids [47] and we have demonstrated that a CB2 agonist provides partial protection against the neurotoxic effects of MDMA [32]. Thus, this increase in IL-1ra levels following neurotoxic MDMA in pretreated animals may contribute to the tolerance observed. In fact it is thought that the relative production of IL-1ra or IL-1β in response to either mild or severe ischemia appears to be quite important in determining outcome [48].

Although IL-1ra may be involved in the expression of tolerance since low dose MDMA pretreatment increases its levels following challenge with neurotoxic MDMA, it is not clear that it is the triggering factor since levels of the antagonist are unaltered at early time points increasing only 96 h after the dose of MDMA. Furthermore, it is not increased 24 h after low dose MDMA, a protocol that also provided neuroprotection.

Low dose MDMA did, however, increase IL-1β levels at an early time point (3 h) and produce changes in the expression of the IL-1 type I receptor. The IL-1R has high affinity for both IL-1β and its antagonist, IL-1ra. Following the action of various proteases, the receptor is cleaved from the membrane and released into the extracellular space. At early time points (3 h and 6 h after low dose MDMA administration) there is an increase in the expression of the receptor in the soluble fraction. The expression normalises at 24 h and increases again at 96 h. Expression of the receptor in the membrane fraction was unaltered at all time points, thus the overall increase may possibly indicate synthesis of the receptor. The expression of the receptor in the soluble fraction represents levels of the proteolytic cleaved extracellular domains of the membrane-bound receptor. This released or soluble form of the receptor, sIL-1RI, binds IL-1β but mediates no signal transduction [25]. Thus, sIL-1RI acts a soluble-decoy receptor for IL-1β, inhibiting the actions of IL-1β at the membrane bound form of IL-1RI. Alongside IL-1ra it attenuates the effects of IL-1β and potentially protects against the deleterious effects of excessive release of the cytokine [49]. Thus, the increase in the soluble form of the receptor 96 h after low dose MDMA in conjunction with the increase in levels of the antagonist may lead to a reduction in the consequences of the elevation of IL-1β levels induced by neurotoxic MDMA. However, similar to IL-1ra, the possible role of this receptor in the tolerance observed is not clear since expression of the soluble form of the receptor is not increased 24 h after low dose MDMA, time at which neuroprotection is observed.

In order to explore the role of IL-1β as a trigger of preconditioning, sIL-1RI was given with the preconditioning dose of MDMA, 96 h before neurotoxic MDMA. This treatment attenuated the protective effect of the low dose MDMA in the ipsilateral side of the frontal cortex having no effect in the contralateral side. Although the soluble receptor also has affinity for IL-1ra [49], the increase in IL-1β levels in the frontal cortex following low dose MDMA and the lack of modification of IL-1ra levels at the same early time points may shift the balance of binding towards the cytokine and away from the antagonist. Therefore, these results suggest a role for IL-1β in the development of tolerance and are supported by studies which describe an important role for NF-κB in ischemic tolerance [50].

Interestingly, the administration of IL-1β directly into the cortex mimicked the preconditioning effect of low dose MDMA. The infusion of the cytokine in the frontal cortex 96 h before neurotoxic MDMA prevented the loss of 5-HT transporter observed after the drug, providing further support for a role of IL-1β in the development of delayed tolerance. Similar preconditioning effects have been observed in other models of damage. Thus, administration of the cytokine directly into the brain induced ischemic tolerance in gerbils [51].

## Conclusions

Low dose MDMA produces delayed preconditioning in rats. The tolerance against neurotoxic MDMA-induced 5-HT transporter loss involves IL-1β. Additional studies are required to identify the role of mediators further downstream in IL-1β induced tolerance against MDMA-induced neurotoxicity.

## Abbreviations

5-HIAA: 5-hydroxyindoleacetic acid; 5-HT: 5-hydroxytryptamine; BSA: bovine serum albumin; IL-1β: interleukin-1beta; IL-1ra: IL-1 receptor antagonist; IL-1RI: IL-1 receptor type I; MDMA: 3,4-methylenedioxymethamphetamine; PBS: phosphate-buffered saline; sIL-1RI: soluble IL-1 receptor type I.

## Competing interests

The authors declare that they have no competing interests.

## Authors' contributions

AM carried out the neurotoxicity studies, immunoassays and Western blot studies and participated in the interpretation of the data, ET participated in the neurotoxicity studies, MDGL participated in the coordination of the study and statistical analysis, MIC participated in the interpretation of the data and helped draft the manuscript, EOS participated in the design and coordination of the study, in the interpretation of the data and in the drafting of the manuscript. All authors read and approved the final manuscript.
